# Computational approaches for *de novo* design and redesign of metal-binding sites on proteins

**DOI:** 10.1042/BSR20160179

**Published:** 2017-03-27

**Authors:** Gunseli Bayram Akcapinar, Osman Ugur Sezerman

**Affiliations:** Department of Statistics and Medical Informatics, School of Medicine, Acibadem University, Istanbul, Turkey

**Keywords:** artificial metalloproteins, computational protein design, computational protein re-design, de novo design, metal binding sites

## Abstract

Metal ions play pivotal roles in protein structure, function and stability. The functional and structural diversity of proteins in nature expanded with the incorporation of metal ions or clusters in proteins. Approximately one-third of these proteins in the databases contain metal ions. Many biological and chemical processes in nature involve metal ion-binding proteins, aka metalloproteins. Many cellular reactions that underpin life require metalloproteins. Most of the remarkable, complex chemical transformations are catalysed by metalloenzymes. Realization of the importance of metal-binding sites in a variety of cellular events led to the advancement of various computational methods for their prediction and characterization. Furthermore, as structural and functional knowledgebase about metalloproteins is expanding with advances in computational and experimental fields, the focus of the research is now shifting towards *de novo* design and redesign of metalloproteins to extend nature’s own diversity beyond its limits. In this review, we will focus on the computational toolbox for prediction of metal ion-binding sites, *de novo* metalloprotein design and redesign. We will also give examples of tailor-made artificial metalloproteins designed with the computational toolbox.

## Introduction

Function of a protein and its interactions with other molecules such as proteins, nucleic acids are determined by protein’s 3D structure. There are over 124000 protein structures deposited in the Research Collaboratory for Structural Bioinformatics (RCSB) Protein Databank (PDB) by June 2016 [1]. In 2012, Yu and colleagues estimated that approximately one-third of the proteins deposited in the PDB database contained metal ions essential for function [2]. In cells, 30–40% of the proteins depend upon at least one metal ion to carry out their biological functions [3,4]. It has been well known that regions or residues of proteins that interact with the metal ligands are very well conserved both in sequence and in structure [5,6].

In many biological systems, proteins that require a metal ion to carry out their physiological function, aka metalloproteins, are very widespread and they perform a variety of functions as storage and transport proteins, enzymes, regulators of gene expression and signal transduction cascade proteins [7–10]. Metalloproteins have been in the focus of the biological research for many years. This intense focus could be traced back to the early X-ray crystallography studies that led to the discovery of presence of a metal atom (iron) along with 3D structure of myoglobin [11,12] and haemoglobin proteins [13].

Metal ions can be crucial for protein structure and function. Proteins are involved in a variety of cellular events and catalytic reactions. Chemical nature of the side chains of amino acids that made up proteins provides them with limited proportion of the chemical functionality seen in nature. On the other hand, association of proteins with cofactors such as small organic molecules, single metal ions or clusters with metal and non-metal atoms granted these proteins with a diversity of functions [12]. Metal ions are involved in nucleophilic catalysis events, in induction of the conformational changes in proteins, in electron transport, in folding and stabilization of protein structures [14]. Metalloproteins play crucial roles in development and progress of a number of diseases including brain diseases such as prion, amyotrophic lateral sclerosis (ALS), Parkinson’s and Alzheimer’s where an effective treatment and cure is still missing [15]. They are also implicated in apoptosis and aging [16,17]. In plants and other microorganisms, they improve metal-adsorption capacities [18]. Well-characterized plant metalloproteins such as metallothioneins and phytochelatins are involved in the uptake of the essential micronutrients required for plant metabolism and detoxification of heavy metal ions [19]. Many metalloproteins in plants are involved in the electron transport chain of the photosynthetic machinery [20,21]. Furthermore, metal ions play a crucial role in protein folding and stability [22–24]. Realization of the importance and involvement of metal-binding sites in a diversity of cellular processes and functions, led to the advancement of various computational tools and algorithms for prediction, identification and engineering of these sites. These advances present us new means to understand the biological function of a protein; to decipher the underlying mechanisms of protein folding and stability and thus improve protein function and stability. An active research area was already established with studies involving *de novo* design, redesign, prediction, optimization and stabilization of protein structure. It has been almost 30 years since the introduction of a helical protein designed from the first principles [25]. In this era, focus is now shifting towards introduction of novel functions to proteins; protein stabilization by *de novo* protein design or redesign; use of metal cofactors. Subsequently, this approach extends the nature’s repository of protein structure and function beyond its natural limits.

In this review, we will focus on the computational tools for the prediction of metal-binding sites on a protein and emphasize the computational tools that are used in *de novo* design and redesign of metal ion-binding sites. We will also highlight some noteworthy artificial metalloproteins that were designed using the developed computational tools.

## Computational tools for *de novo* design and redesign of metalloproteins

Unprecedented increase in the number of protein sequences and structures deposited in the public databases due to advances in genome-sequencing technologies and experimental methods for structure determination, poses a great challenge for researchers in terms of prediction of the biological functions of the deposited proteins. Since metal ions play crucial roles in many biological processes, their presence in protein structures reveals essential information about protein’s inherent function(s). Owing to the fact that determination of structural and functional features of a protein such as metal ion-binding sites using experimental methods is still challenging because of the problems related to cost, time and automation of processes, there is an increasing demand for prediction of those features using computational methods. Use of computational methods for the prediction of metal ion-binding sites not only contributes to expansion of the existing knowledgebase, but also aids *de novo* metalloprotein design and redesign by providing necessary structural information about the metal co-ordination environment. This information is used in the design of tailored and stable metalloproteins with improved functions. Therefore, prosperity of a metalloprotein design largely depends on the quantity and quality of available information on metal ion-binding sites, protein scaffolds and physiochemical rules that direct the folding of a polypeptide into a functional protein [26,27].

### Computational prediction of metal ion-binding sites

**Figure 1:**
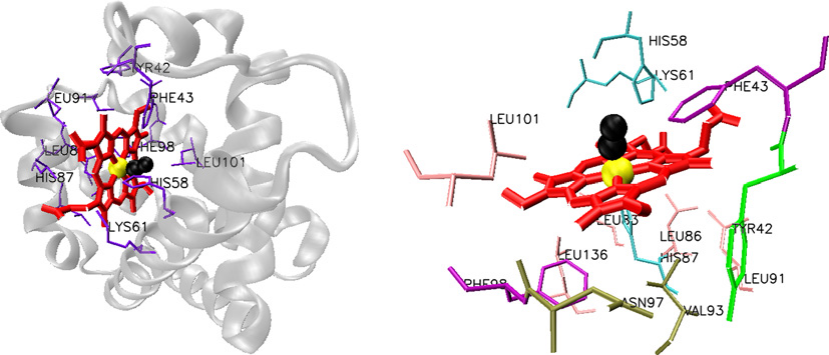
Structure of chain C of cross-linked carbonmonoxy haemoglobin A from *Homo sapiens* (left) Haem-coordinating residues are shown on the right. Haem group (red) with bound Fe (yellow) and carbonmonoxide (black). PDB ID: 1SDK.

Metal ion-binding sites in proteins exhibit a wide range of diversity. In some proteins, backbone oxygen and nitrogen atoms are involved in metal ion binding, whereas in some proteins side chain oxygen, nitrogen and sulfur atoms are involved. Metal ions were also found to be selective in their binding to their respective ligands. In a 2012 paper, Yu and colleagues used 1109 metal ion-binding polypeptides and predicted the metal ion-binding sites and verified that metal ions preferentially bind to certain residues on the protein [2]. For Ca^+2^, favoured amino acids were predicted to be D, E, N and G; for Cu^+2^ H; for Mg^+2^ D and E; for Fe^+3^ H, D, E, C and Y. Residues that reside partially within 3.5 Å of the metal ion were considered as metal ion-binding residues. As the metal ion-binding sites diversify, correct identification of the ligands in metal ion-binding sites with experimental methods becomes more problematic without prior knowledge of the identity of the bound metal ion since current high-throughput methods based on X-ray absorption spectroscopy can only identify its presence but cannot identify the residues involved in metal ion binding [28,29]. Besides, most of the computational tools for metal ion-binding site prediction use the information mostly derived from crystals of the metal ion-bound form of the proteins, holo (metal ion-bound) forms whereas a majority of the recently resolved structures are from the apo (metal ion-free) forms [30]. Moreover, metal ion binding is a dynamic process that often results in structural rearrangements of the residues in binding pockets [31,32]. Additionally, in some cases, a metal ion is buried in a prosthetic group, tightly bound, specific non-polypeptide unit required for the biological function of some proteins, such as haem that consists of a central Fe (II/III) atom in a protoporphyrin ring [13] as in haemoglobin structure (Figure 1). Consequently, prediction and identification of metal ion-binding sites pose a much more complex problem that cannot be solved solely based on simple geometrical criteria or sequence information. Therefore, different computational methods and algorithms have been introduced for the prediction of metal ion-binding sites and the residues involved in this process. Moreover, prediction of most probable sites of metal ion-binding motifs in a newly identified protein would facilitate redesign of that site to better accommodate a metal to modify its structural and functional properties. After prediction and comparison with known motifs, this knowledge would enable establishment of a metal ion-binding site library that could be utilized for protein engineering purposes.

Most computational methods rely on the sequence information and they target the identification of metal-binding motif or motifs in well characterized metal ion-binding proteins. These motifs are then used to search for similar patterns in newly identified proteins. Machine learning methods have also been introduced to the problem only recently [7,33–37]. Programs such as MetalPredator [38], MetalDetector v2.0 [39], SeqCHED Server [34], ZincFinder [35], SVMProt [36,37] employ sequence based approaches for the prediction of the metal ion-binding sites. For instance MetalDetector v2.0 [39] uses cysteine and histidine residues and therefore focuses only on transition metals, haem and Fe/S clusters for the prediction, by predicting the metal-bonding state of these residues and number of bound metal ions. On the other hand, MetalPredator [38] utilizes two libraries of Hidden Markov Model profiles representative of Pfam domains and structural motifs that bind Fe/S clusters. Some prediction software such as FINDSITE-metal [40], mFASD [41], TEMSP [42], FEATURE metal scanning [43–45] utilize structural information for prediction of the metal ion-binding sites. A few of them such as MetSite [46], 3DLigandSite [47], Fragment Transformation Method [2] combine information from both protein sequence and structure. Use of *ab initio* methods as in IonCom [48] server has also been recently realized. Several helper tools such as MetalS^2^ [49], MetalS^3^ [50], FindGeo [51], CheckMyMetal (CMM) [52] were developed for pairwise structural alignment, database mining of metal-binding sites, determination of metal co-ordination geometry, validation of metal-binding sites in PDB structures respectively. Software developed for the prediction of metal ion-binding sites, their rationale and web links are summarized in Table 1. Although these servers provide a large selection of different algorithms for the prediction, some of them are only limited to the prediction of a single metal (i.e. MetalPredictor and MetalDetectorv2.0 only predict Fe–S cluster-binding sites whereas FEATURE metal scanning tool, TEMSP, ZincFinder predict Zn^2+^-binding sites, the rest of the servers provide a diverse range of metals that are associated with metalloproteins). Databases such as MetalPDB [4], MetalMine [53], Metal Interactions in Protein Structures (MIPS) [54], MESPEUS_10 [55], COMe [56], MetLigDB [57] as listed in Table 2, encompass information on both sequence motifs that define metal ion-binding sites and structure of metal ions and their corresponding residues that are deposited in various sequence and structural databases.

**Table 1:** Software designed for metal ion-binding site prediction

Software	Short Description	Ref.	Link
**FINDSITE-metal**	Sequence and structure based	[40]	http://cssb.biology.gatech.edu/findsite-metal
**MetalPredator**	Sequence based	[38]	http://metalweb.cerm.unifi.it/tools/metalpredator/
**MetalDetector v2.0**	Sequence based	[39]	http://metaldetector.dsi.unifi.it/v2.0/
**SeqCHED Server**	Sequence based	[34]	http://ligin1.weizmann.ac.il/∼ronenle/Web/SeqCHED/
**FEATURE metal scanning**	Structure based	[43–45]	http://feature.stanford.edu/metals/
**MetSite**	Sequence and structure based	[46]	http://bioinf.cs.ucl.ac.uk/structure/
**mFASD**	Structure based	[41]	source code: http://staff.ustc.edu.cn/~liangzhi/mfasd/
**TEMSP**	Structure based	[42]	http://netalign.ustc.edu.cn/temsp/
**MetalS^2^**	Structure based	[49]	http://metalweb.cerm.unifi.it/tools/metals2/
**MetalS^3^**	Structure based	[50]	http://metalweb.cerm.unifi.it/tools/metals3/
**FindGeo**	Structure based	[51]	http://metalweb.cerm.unifi.it/tools/findgeo/
**CMM**	Structure based	[52]	http://csgid.org/csgid/metal_sites/
**ZincFinder**	Sequence based	[35]	http://zincfinder.dsi.unifi.it
**IonCom**	Sequence and structure based	[48]	http://zhanglab.ccmb.med.umich.edu/IonCom/
**SVMProt**	Sequence based	[37,36]	http://bidd2.nus.edu.sg/cgi-bin/svm-prot/svmprot.cgi
**3DLigandSite**	Sequence and structure based	[47]	http://www.sbg.bio.ic.ac.uk/3dligandsite/

**Table 2:** List of databases for metalloproteins, metal ion-binding site motifs and structural information on metal ion-binding sites

Databases		ReferenceLink
**MetalPDB**	[4]	http://metalweb.cerm.unifi.it/
**MetalMine**	[53]	http://metalmine.naist.jp/metalmine009/index.html
**MIPS**	[54]	http://dicsoft2.physics.iisc.ernet.in/mips/
**MESPEUS_10**	[55]	http://mespeus.bch.ed.ac.uk/MESPEUS_10/
**COMe**	[55,56]	http://www.flymine.org/come/
**MetLigDB**	[57]	http://silver.sejong.ac.kr/MetLigDB

### Computational design tools, strategies for *de novo* design and redesign of metalloproteins

Many of the biological and chemical reactions that establish the foundations of life such as water oxidation, carbon dioxide reduction, nitrogen fixation, photosynthesis require involvement of metalloproteins or metalloenzymes [58]. Chemical transformations achieved by metalloproteins are diverse [59]. Despite the diversity of these reactions, we are still far from completely understanding the principles that govern these metalloproteins when performing their functions. Much effort has been put to fully mimic or exploit these processes. As *de novo* protein design and redesign methods have been advancing for the past decades, application of these methods to artificial metalloprotein and metalloenzyme design and redesign is inevitable. Previous research has created fully functional *de novo* designed metalloproteins that exhibit activity for a variety of reactions ranging from ester and organophosphate hydrolysis [60,26] to nitric oxide reduction [60,61].

In metalloproteins, metal ions either interact with amino acid side chains or accompanying residues. Most of the metal-binding sites are known to be promiscuous and can accommodate non-native metals with similar properties [62]. Redesign of metalloproteins usually involves modification of a well-characterized and well-known native protein through engineering or introduction of metal-binding ligands to protein structure to accommodate non-native metals. For *de novo* design of metalloproteins, protein structures should be designed from scratch starting from the amino acid sequences. Residues for metal ion binding should also be introduced to the structure [63]. Both methods require correctly folded and functional final protein structures with introduced metal ions or cofactors. *De novo* design approach is more complex since physiochemical rules that drive the protein folding [64] have not yet been deciphered completely. Moreover, “inverse folding problem” adds another layer to this complexity. *De novo* protein design uses the “inverse folding problem”, which states that different combinations of amino acid sequences can fold into the same 3D structure and resulting proteins can perform the same function [65,66]. In protein design, we start with a rigid or flexible backbone structure and try to determine a sequence that would fold into this structure. As a variety of sequences fold into the same structure, there is degeneracy. Therefore, the accuracy and availability of the template structures for protein design has a huge affect in the success of the final design.

**Figure 2:**
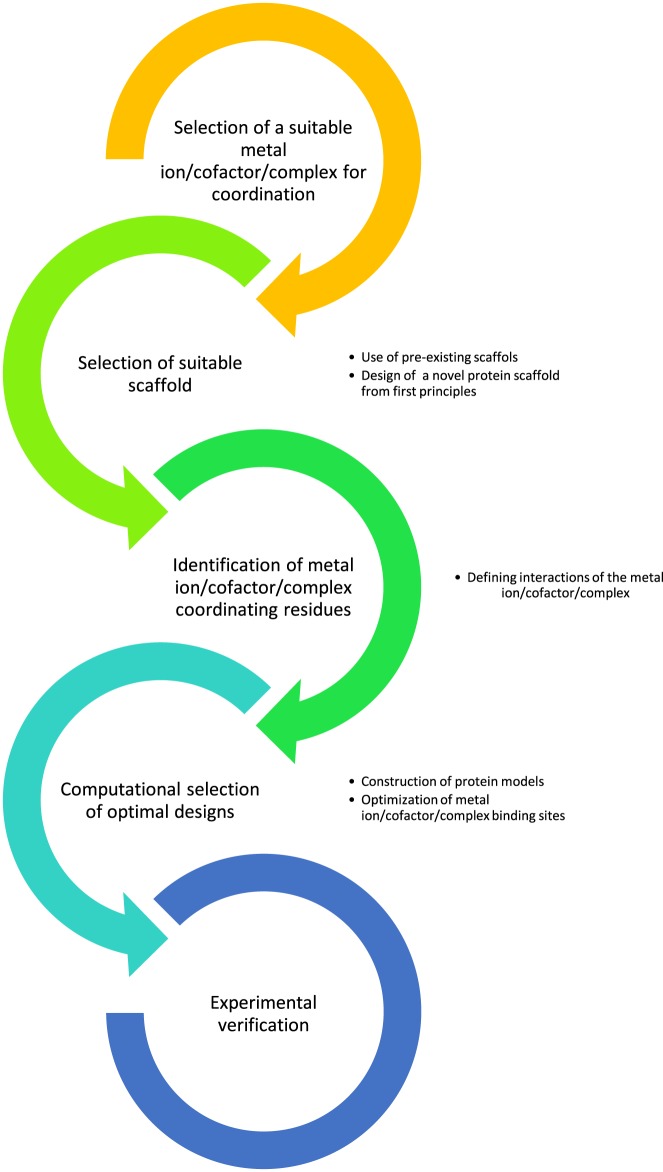
A typical workflow for *de novo* metalloprotein design and redesign

The current collection of metalloproteins is largely coming from nature’s own repository. This repository is now being diversified by *de novo* design and redesign approaches with the aid of the knowledgebase of metal ion-binding sites. A typical workflow for this procedure is shown in Figure 2. Successful application of *de novo* design and redesign approaches create a diversity of advanced proteins with either new or modified functions. Additionally, through the application of these methods, an excellent platform is formed to study structure–function relationship and protein folding problem further.

Construction of artificial or redesigned metalloproteins requires detailed information on the nature of the metal ion-binding motifs, how the metal ions are co-ordinated (ligand–metal interactions and binding pocket geometry, geometrical arrangements, redox states) and finally a detailed information on the structure–function of the scaffold protein that would accommodate the metal ion. This, in turn, requires the use of state of the art computational tools for design and experimental techniques for analysis and characterization. A variety of deterministic, stochastic and probabilistic methods have been applied for *de novo* protein design [67]. With the recent advances in the computational field (high performance computing, better algorithms, faster processors), backbone flexibility is also incorporated into the calculations [68].

A compelling number of programs such as Protein WISDOM [68], HostDesigner [69], METALSEARCH [70], CORE [71], RosettaDesign [72], EvoDesign [73], RosettaBackrub [74], IPRO (OptGraft procedure) [75], PyRosetta [76] has been in use for *de novo* protein design (Table 3). These software provide *in silico* workbenches for the design of novel, stable and functional proteins. Most of these programs use force fields to represent molecular interactions and to search for sequences with lowest energy in the target fold since the minimum free energy conformation of a protein is considered as the native fold of the protein.

**Table 3:** Programs for *de novo* protein design

Software		Ref.Link
**METALSEARCH**	[70]	Not available
**Protein WISDOM**	[68]	http://www.proteinwisdom.org
**HostDesigner**	[69]	https://sourceforge.net/projects/hostdesigner-v3-0/
**CORE**	[71]	Not available
**RosettaDesign**	[72]	http://rosettadesign.med.unc.edu
**RosettaBackrub**	[74]	https://kortemmelab.ucsf.edu/backrub/cgi-bin/rosettaweb.py?query=index
**EvoDesign**	[73]	http://zhanglab.ccmb.med.umich.edu/EvoDesign
**IPRO (OptGraft)**	[75]	http://www.maranasgroup.com/submission/ipro2014.htm
**PyRosetta**	[76]	http://www.pyrosetta.org/

Although all of them have been used for *de novo* design of metalloproteins, only three of them, HostDesigner, METALSEARCH and IPRO (OptGraft) are specific for *de novo* design or redesign of metalloproteins. HostDesigner is a combinatorial chemistry-based program that uses two complementary algorithms LINKER and OVERLAY for creation of a number of candidate structures and to discover metal ion receptors in these structures [69]. Two user-defined complex fragments are connected with linking fragments deposited in the library and structures are built by LINKER. Next, a single user-defined complex structure is built by OVERLAY through the superimposition of the linking fragments on to this structure.

METALSEARCH, being one of the earliest design programs, creates lists of four residues that might form tetrahedral metal ion-binding sites if amino acids in the native protein were replaced by cysteine or histidine residues. Program also provides dihedral angles of the amino acids and the co-ordinates of the predicted metal ion as output [70]. OptGraft is a computational program that is present in IPRO suite of programs and it is specifically designed for transferring a binding site on to an existing protein scaffold. For this purpose, possible binding pocket placement combinations are exhaustively explored using mixed-integer linear optimization and a ranked list of possible designs that fit the geometric criteria and orientation of the native binding pocket is generated. Moreover, this procedure also assesses the impact of the new pocket on the protein structure and if there is a potential distortion in the structure, small mutations in the neighbouring residues are introduced to counteract their probable distortional effects [75].

#### Applications

##### Computational design of novel redox centres

METALSEARCH was used for *de novo* design of a rubredoxin-like Fe site [ 77], for the introduction of zinc-binding sites to the designed four helix bundle protein α4 and to the Ig-binding B1 domain of the Streptococcal protein G [ 78, 79]. It has been long known that iron ion in rubredoxin proteins is co-ordinated by four cysteine residues. Farinas et al. [ 77] designed a redox centre that mimics rubredoxin’s into the Ig-binding B1 domain of the Streptococcal protein G by taking into account the backbone movements while maintaining the structural integrity and stability of the B1 protein. For this purpose, they first used METALSEARCH to identify potential metal ion-binding sites that are capable of tetrahedral co-ordination in the B1 protein structure. Contrary to previous work of the same group [ 78, 79], they made an attempt to use a more realistic backbone flexibility description in the computational part. As an input to the METALSEARCH, rather than using the averaged NMR structure, researchers selected 6 randomly calculated NMR structures out of 60 that reflected a more realistic backbone flexibility. Co(II) and Cd(II) bound designed variants’ stability and integrity were confirmed with spectroscopic methods. Fe(II) complex of the designed variant was produced and analysis and characterization of the rubredoxin centre revealed that the mutant rubredoxin centre mimicked oxidized rubredoxin. Nanda et al. [ 80] also managed to design a redox active minimal β protein scaffold (RM1) that exhibit rubredoxin activity through formation of a stable, redox-active 4-Cys thiolate Fe(II/III) site. The designed complex was further shown to be stable through repeated cycles of oxidation and reduction even in the presence of an oxygen containing environment.

**Figure 3:**
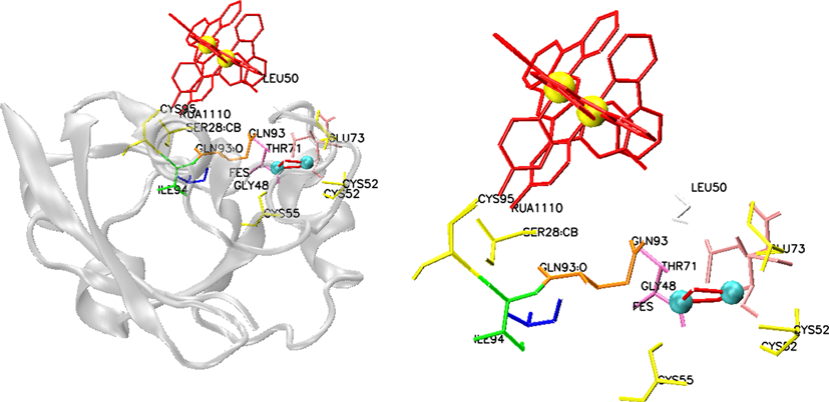
Structure of Ru(bpy)2(mbpy)-modified bovine adrenodoxin protein (left) Ru(bpy)2 complex (red) is covalently bound to adrenodoxin via Cys^95^. An iron–sulfur cluster (cyan-red) is also present in the protein. Ruthenium is shown in yellow (PDB ID: 2BT6). Ru(bpy)2 complex (red) and iron–sulfur cluster (red-cyan) co-ordinating residues are shown on the right.

In another design approach, computationally designed metalloprotein using an in-house developed CORE software [71] that functions as an artificial redox centre was produced with the ability to mimic photosynthesis [ 81]. An antiparallel four helix bundle containing two helix–loop–helix peptides connected through a disulfide bridge was used as the scaffold. This scaffold is functionalized through histidine-coordinated Ruthenium(II) Bipyridine (Ru(bpy)2) and haem cofactors by engineering of the binding sites. The exterior hydrophilicity of the bundle was provided by the salt bridges formed primarily between glutamic acid and lysine residues, whereas identity of the hydrophobic core residues inside the helices were determined using the CORE software. An exemplary structure of Ru(bpy)2(mbpy)-modified bovine adrenodoxin protein was shown in Figure 3 to show the metal co-ordination. The backbone structure that was used as an input to the software contains alanine in all positions with the exception of the exterior hydrophilic residues and cofactor-coordinating residues. Software predicts the optimum hydrophobic core residues based on the protein thermal stability and co-operativity. Consequently, the resulting protein mimics photosynthesis following photo-excitation participating in multiple oxidation and reduction cycles with exogenous electron acceptors and donors.

##### Computational design of artificial metalloproteins and metalloenzymes

Computational protein design software Rosetta Design, is a highly versatile tool that can be exploited for analysis of protein–metal interactions in the design of artificial metalloproteins. Recently, this software was used to improve the catalytic performance of an artificially designed metalloenzyme and to design a promiscuous metalloprotein where an incorporated unnatural amino acid (2,2′-bipyridin-5yl) alanine (Bpy–Ala) residue co-ordinates the metal ion with octahedral co-ordination geometry [80,81]. Heinisch et al. [82] adopted a computational design approach for the genetic optimization of a first-generation artificial metalloenzyme using this software. Previous research produced an assembly of Noyori-type pianostool complex [(5-Cp*)Ir(pico)Cl] in a native human carbonic anhydrase II enzyme that is capable of asymmetric transfer hydrogenation of a salsolidine precursor (a cyclic imine) with moderate enantioselectivity [83] and combinations of these first-generation artificial metalloenzymes with naturally occurring enzymes were also successfully utilized in redox cascade reactions [84]. The crystal structure of the complex revealed that the metal-coordinating moiety was not fully occupied, which led to a modest activity and selectivity. Therefore, RosettaDesign [72] was used to improve cofactor-binding sites and the second co-ordination sphere around cofactor in the first generation artificial metalloenzymes. The software produced four scaffold variants that have mutations for protein backbone stabilization and that enable hydrophobic cofactor burial. The produced designs not only exhibited increased affinity towards the cofactor but also increased activity, turnover number and enantioselectivity with respect to the first-generation artificial metalloenzyme. Mills et al. [85] employed a similar design approach with the use of RosettaMatch algorithm [86] and RosettaDesign [72] for the co-ordination of metal ions by a metalloprotein through the use of unnatural amino acids. For this purpose, an active site capable of catalysing oxidative ring opening of catechol substrates was designed. Four theoretical enzyme active sites, also referred as “theozymes” were constructed including unnatural amino acid Bpy–Ala, catechol containing dopamine molecule, a tyrosine and a histidine ligand for metal co-ordination. RosettaMatch was adopted for the identification of a set of backbone positions in native scaffolds that could accommodate theozyme geometrical restraints. RosettaDesign was then employed to introduce additional stabilizing interactions for the Bpy–Ala complex to the RosettaMatch output. An additional filtering step was applied to find the optimum structures. Although 13 genes that encode artificial metalloproteins are designed and synthesized, only five of the designed proteins yielded soluble, full length proteins in the presence of the unnatural amino acid. These proteins were shown to be promiscuous and bind to divalent cations including Co^2+^, Zn^2+^, Fe^2+^ and Ni^2+^. Moreover, structural analysis via X-ray crystallography revealed that the designed proteins exhibit only slight deviations from the designed models.

Additionally, the OptGraft procedure in IPRO software suite was used by Fazelinia et al. [75] to graft a calcium-binding pocket of thermitase protein to the first domain of CD2 protein (a rat cell adhesion protein that does not bind calcium). Minimally perturbed structures were created by identification of suitable locations for the grafting using OptGraft procedure. Candidate residues that would ensure the desired spatial restraints for the binding pocket were determined using CHARMM energy calculations. Designs with higher stability were further selected. Novel CD2 variants were shown to exhibit higher affinities for terbium and selective binding to calcium.

Researchers also developed in-house programs for the *de novo* design of metal ion-binding sites. Zhu et al. [ 87] managed to incorporate computationally engineered zinc-binding sites to *de novo* designed DS119 protein with βαβ fold with 1:1 stoichiometry using an in-house developed program. Likewise, Yeung et al. [88] used myoglobin as scaffold to introduce nitric oxide reductase activity by grafting three histidines and one glutamate residues that would accommodate a haem and non-haem Fe_B_ centre. They followed a molecular dynamics (MD)-based modelling approach for the *in silico* introduction of mutations to myoglobin scaffold and to evaluate the ability of the designed metal ion-binding site to bind an iron ion. To achieve this three *in silico* mutations (L29H, F43H and V68E) were introduced into sperm whale myoglobin structure using the VMD software suite. Additionally, molecular dynamics simulations (NVT ensemble, 310 K, 10000 steps, 1 fs/step) with NAMD software were performed to estimate the ability of the mutant protein to bind Fe atom through the engineered metal ion-binding site. The triple mutant of the sperm whale myoglobin was constructed and mutant protein was expressed in *Escherichia coli*. The mutant protein was further purified and characterized. Finally, the tailor-made protein was shown to exhibit nitric oxide reductase activity.

##### Computational design of metal-templated interfaces

In 2010, Salgado et al. used Metal Templated Interface Redesign (MeTIR) approach to gain insights about the role played by metal co-ordination via metal co-ordinated nucleation process in the formation of early protein folds and complexes throughout evolution [89]. For this purpose, they have redesigned a monomeric protein, namely cytochrome *cb*_562_ with non-self-associating surface and haem groups in its structure to obtain a variant that exhibits Zn co-ordinated self-association properties. A second redesign step was introduced to Zn co-ordinated D2-symmetrical tetramer to engineer the complex further into a complex that exhibits self-assembly in the presence or absence of metals. Mutations were introduced only into the residues on the surface of cytochrome *cb*_562_ to allow easy tracing of the structure with crystallographic methods and to preserve the overall fold. Residues inside the protein (with low solvent accessible surface are (SASA) values), residues contacting heme groups or Zn atoms, residues that were involved in side chain-main chain H-bonding interactions remained and they were not selected for mutations. A variant of RosettaDesign algorithm was employed for the rotamer optimization of the selected residues to find minimal number of mutations that make a maximum impact towards the self-assembly of the monomeric protein. The energy and packing scores obtained, ranked and position of candidate residues with respect to the undesirable residues were evaluated to find optimal residues at the interface for redesign.

##### Knowledge-driven metalloprotein designs

In some cases, computational programs were not used for the design. Instead, designs were based on previous information derived from sequence, structure and function of proteins, previous literary knowledgebase and intuition of the researchers [90–92]. In most of the cases, previously designed protein scaffolds are modified to engineer the binding pockets [63, 88, 91, 93–96]. In one recent case, noble metals were introduced to iron-binding sites of the haem proteins such as myoglobin and the designed protein was subjected to directed evolution [97]. First, apo-form of haem proteins were expressed in *E. coli* using minimal media devoid of Fe for the minimization of haemin biosynthesis, at low temperature to alleviate the instability of the apo-form. Myoglobin from *Physeter macrocephalus* and cytochrome P450 BM3h from *Bacillus megaterium* were found to be overexpressed with or without mOCR stability tags in relatively high yields. These haem lacking apo-forms were purified and holo-forms of the proteins were reconstituted with noble metal-porphyrin IX cofactors. Next, eight myoglobin variants with mutations in the axial ligand position (His^93^) were expressed in apo-form and then reconstituted with various noble metal-porphyrin IX cofactors. Consequently, the resulting proteins had the capability to catalyse the functionalization of C–H bonds to form C–C bonds by carbene insertion and add carbenes to both β-substituted vinylarenes and unactivated aliphatic α-olefins.

Another approach involves the use of biotin–streptavidin (Sav) system to anchor metal complexes to a specific location in a protein [59 ,98–100]. This methodology was applied to artificial cupredoxin synthesis recently [59]. A systematic modulation of the primary and secondary co-ordination spheres of artificial copper proteins was performed. Biotinylated copper complexes within a cysteine containing variant of Sav have exhibited properties similar to native cupredoxins. Fine tuning of the position of the Cu centres inside the modified Sav was achieved by changing length of the linker between biotin and the copper complex. A similar approach was employed by Heinisch et al. [82] and Quinto et al. [98] in the design of an artificial metalloenzyme that exhibits activity as an NAD(P)H dependent transfer hydrogenase recently. To this end, a biotinylated iridium cofactor was introduced to variants of Sav scaffold that resulted in the successful activity of the artificial metalloenzymes.

## Conclusions and future perspectives

Metalloproteins advanced through millions of years of protein evolution. Considering this huge evolutionary history, we are still far from perfecting our *de novo* design attempts. Computational design offers an alternative but complementary route to directed evolution experiments for the introduction of novel functionalities. Learning from nature’s toolbox and rules that drive protein evolution, we can improve our designs [95]. Considerable efforts have been put for the design and production of new artificial metalloproteins [85]. However, there are still challenges such as achieving accuracy at the atomic level since flexible polar amino acid side chains form metal-binding sites through alternative ways. Computational design principles can be used to design a library of variants that could co-ordinate a metal cofactor. Additionally, it can be used to reduce number of the variants for experimental characterization [82]. Initially, the metalloprotein design was focused on the design and formation of mono-metal centres on the designed proteins. This horizon was expanded by the introduction of di-metal centres [91, 101] and current research is focused on building multi-metal cluster containing artificial metalloproteins [58] and specifically metalloenzymes since most remarkable and complex chemical transformations are achieved through the use of multi-metal centres [59]. As these horizons expand, design of different scaffolds from first principles that would host different metal centres is also needed. Moreover, directed evolution should complement *de novo* protein design efforts to produce artificial metalloproteins with improved properties. Although an increasing number of graphical user interfaces for protein design and metal ion-binding site prediction are being developed, use of computational programs for the design of artificial metalloproteins does not follow the same pace. Therefore, there is an emerging need to develop user-friendly tools specific for metalloprotein design and engineering.

## Abbreviations

BIM: bioinorganic motif
bpy: bipyridine
Bpy–Ala: (2,2′-bipyridin-5yl) alanine
CHARMM: Chemistry at Harvard Macromolecular Mechanics
CMM: Check My Metal
fs: femtosecond
MIPS: metal interactions in protein structures
PDB: Protein Databank
Ru(bpy)2: ruthenium(II) bpy
Sav: Streptavidin
SASA: Solvent Accessible Surface Area

## References

[1] Rose P.W., Bi C., Bluhm W.F., Christie C.H., Dimitropoulos D., Dutta S. (2013) The RCSB Protein Data Bank: new resources for research and education. Nucleic Acids Res. 41, D475–D482 23193259 10.1093/nar/gks1200 PMC3531086

[2] Lu C.-H., Lin Y.-F., Lin J.-J. and Yu C.-S. (2012) Prediction of metal ion-binding sites in proteins using the fragment transformation method. Plos ONE 7, e39252 22723976 10.1371/journal.pone.0039252 PMC3377655

[3] Andreini C., Bertini I. and Rosato A. (2009) Metalloproteomes: a bioinformatic approach. Acc. Chem. Res. 42, 1471–1479 19697929 10.1021/ar900015x

[4] Andreini C., Cavallaro G., Lorenzini S. and Rosato A. (2013) MetalPDB: a database of metal sites in biological macromolecular structures. Nucleic Acids Res. 41, D312–D319 23155064 10.1093/nar/gks1063 PMC3531106

[5] Kasampalidis I.N., Pitas I. and Lyroudia K. (2007) Conservation of metal-coordinating residues. Proteins 68, 123–130 17393459 10.1002/prot.21384

[6] Opella S.J., DeSilva T.M. and Veglia G. (2002) Structural biology of metal-binding sequences. Curr. Opin. Chem. Biol. 6, 217–223 12039007 10.1016/s1367-5931(02)00314-9

[7] Brylinski M. and Skolnick J. (2011) FINDSITE-metal: Integrating evolutionary information and machine learning for structure-based metal binding site prediction at the proteome level. Proteins 79, 735–751 21287609 10.1002/prot.22913 PMC3060289

[8] Barondeau D.P. and Getzoff E.D. (2004) Structural insights into protein-metal ion partnerships. Curr. Opin. Struct. Biol. 14, 765–774 15582401 10.1016/j.sbi.2004.10.012

[9] Yamashita M.M., Wesson L., Eisenman G. and Eisenberg D. (1990) Where metal ions bind in proteins. Proc. Natl. Acad. Sci. U.S.A. 87, 5648–5652 2377604 10.1073/pnas.87.15.5648 PMC54384

[10] Holm R.H., Kennepohl P. and Solomon E.I. (1996) Structural and functional aspects of metal sites in biology. Chem. Rev. 96, 2239–2314 11848828 10.1021/cr9500390

[11] Kendrew J.C., Bodo G., Dintzis H.M., Parrish R.G., Wyckoff H. and Phillips D.C. (1958) A three-dimensional model of the myoglobin molecule obtained by X-ray analysis. Nature 181, 662–666 13517261 10.1038/181662a0

[12] Finkelstein J. (2009) Metalloproteins. Nature 460, 813 19675640 10.1038/460813a

[13] Perutz M.F., Muirhead H., Cox J.M. and Goaman L.C.G. (1968) Three-dimensional Fourier synthesis of horse oxyhaemoglobin at 2.8 Å resolution: the atomic model. Nature 219, 131–139 5659637 10.1038/219131a0

[14] Regan L. (1995) Protein design: novel metal-binding sites. Trends Biochem. Sci. 20, 280–285 7667881 10.1016/s0968-0004(00)89044-1

[15] Gaggelli E., Kozlowski H., Valensin D. and Valensin G. (2006) Copper homeostasis and neurodegenerative disorders (Alzheimer’s, prion, and Parkinson’s diseases and amyotrophic lateral sclerosis). Chem. Rev. 106, 1995–2044 16771441 10.1021/cr040410w

[16] Liu J., Chakraborty S., Hosseinzadeh P., Yu Y., Tian S., Petrik I. (2014) Metalloproteins containing cytochrome, iron-sulfur, or copper redox centers. Chem. Rev. 114, 4366–4469 24758379 10.1021/cr400479b PMC4002152

[17] Lothian A., Hare D.J., Grimm R., Ryan T.M., Masters C.L. and Roberts B.R. (2013) Metalloproteomics: principles, challenges and applications to neurodegeneration. Front. Aging Neurosci. 5, 35 23882215 10.3389/fnagi.2013.00035 PMC3714543

[18] Fosso-Kankeu E. and Mulaba-Bafubiandi A.F. (2014) Implication of plants and microbial metalloproteins in the bioremediation of polluted waters: a review. Phys. Chem. Earth Parts A/B/C 67–69, 242–252

[19] Cobbett C. and Goldsbrough P. (2002) Phytochelatins and metallothioneins: roles in heavy metal detoxification and homeostasis. Annu. Rev. Plant Biol. 53, 159–182 12221971 10.1146/annurev.arplant.53.100301.135154

[20] Yruela I. (2013) Transition metals in plant photosynthesis. Metallomics 5, 1090–1109 23739807 10.1039/c3mt00086a

[21] Merchant S. and Dreyfuss B.W. (1998) Posttranslational assembly of photosynthetic metalloproteins. Annu. Rev. Plant Physiol. Plant Mol. Biol. 49, 25–51 15012226 10.1146/annurev.arplant.49.1.25

[22] Botelho H.M., Koch M., Fritz G. and Gomes C.M. (2009) Metal ions modulate the folding and stability of the tumor suppressor protein S100A2. FEBS J. 276, 1776–1786 19267779 10.1111/j.1742-4658.2009.06912.x

[23] Sujak A., Sanghamitra N.J.M., Maneg O., Ludwig B. and Mazumdar S. (2007) Thermostability of proteins: role of metal binding and pH on the stability of the dinuclear Cu(A) site of *Thermus* *thermophilus*. Biophys. J. 93, 2845–2851 17604317 10.1529/biophysj.106.101162 PMC1989708

[24] Palm-Espling M.E., Niemiec M.S. and Wittung-Stafshede P. (2012) Role of metal in folding and stability of copper proteins *in vitro*. Biochim. Biophys. Acta 1823, 1594–1603 22306006 10.1016/j.bbamcr.2012.01.013

[25] Regan L. and DeGrado W. (1988) Characterization of a helical protein designed from first principles. Science 241, 976–978 3043666 10.1126/science.3043666

[26] Lu Y., Yeung N., Sieracki N. and Marshall N.M. (2009) Design of functional metalloproteins. Nature 460, 855–862 19675646 10.1038/nature08304 PMC2770889

[27] Li X., Zhang Z. and Song J. (2012) Computational enzyme design approaches with significant biological outcomes: progress and challenges. Comput. Struct. Biotechnol. J. 2, e201209007 24688648 10.5936/csbj.201209007 PMC3962085

[28] Passerini A., Lippi M. and Frasconi P. (2012) Predicting metal-binding sites from protein sequence. IEEE/ACM Trans. Comput. Biol. Bioinform. 9, 203–213 21606549 10.1109/TCBB.2011.94

[29] Shi W., Punta M., Bohon J., Sauder J.M., D’Mello R., Sullivan M. (2011) Characterization of metalloproteins by high-throughput X-ray absorption spectroscopy. Genome Res. 21, 898–907 21482623 10.1101/gr.115097.110 PMC3106322

[30] Babor M., Gerzon S., Raveh B., Sobolev V. and Edelman M. (2008) Prediction of transition metal-binding sites from apo protein structures. Proteins 70, 208–217 17657805 10.1002/prot.21587

[31] Asante-Appiah E., Seeholzer S.H. and Skalka A.M. (1998) Structural determinants of metal-induced conformational changes in HIV-1 integrase. J. Biol. Chem. 273, 35078–35087 9857042 10.1074/jbc.273.52.35078

[32] de Peredo A.G., Saint-Pierre C., Latour J.-M., Michaud-Soret I. and Forest E. (2001) Conformational changes of the ferric uptake regulation protein upon metal activation and DNA binding; first evidence of structural homologies with the diphtheria toxin repressor. J. Mol. Biol. 310, 83–91 11419938 10.1006/jmbi.2001.4769

[33] Liu T. and Altman R.B. (2009) Prediction of calcium-binding sites by combining loop-modeling with machine learning. BMC Struct. Biol. 9, 72 20003365 10.1186/1472-6807-9-72 PMC2808310

[34] Levy R., Edelman M. and Sobolev V. (2009) Prediction of 3D metal binding sites from translated gene sequences based on remote-homology templates. Proteins 76, 365–374 19173310 10.1002/prot.22352

[35] Passerini A., Andreini C., Menchetti S., Rosato A. and Frasconi P. (2007) Predicting zinc binding at the proteome level. BMC Bioinformatics 8, 39 17280606 10.1186/1471-2105-8-39 PMC1800866

[36] Cai C.Z., Han L.Y., Ji Z.L., Chen X. and Chen Y.Z. (2003) SVM-Prot: web-based support vector machine software for functional classification of a protein from its primary sequence. Nucleic Acids Res. 31, 3692–3697 12824396 10.1093/nar/gkg600 PMC169006

[37] Lin H., Han L., Zhang H., Zheng C., Xie B., Cao Z. (2006) Prediction of the functional class of metal-binding proteins from sequence derived physicochemical properties by support vector machine approach. BMC Bioinformatics 7, S13 10.1186/1471-2105-7-S5-S13 PMC1764469 17254297

[38] Valasatava Y., Rosato A., Banci L. and Andreini C. (2016) MetalPredator: a web server to predict iron-sulfur cluster binding proteomes. Bioinformatics 32, 2850–2852 27273670 10.1093/bioinformatics/btw238

[39] Passerini A., Lippi M. and Frasconi P. (2011) MetalDetector v2.0: predicting the geometry of metal binding sites from protein sequence. Nucleic Acids Res. 39, W288–W292 21576237 10.1093/nar/gkr365 PMC3125771

[40] Brylinski M. and Skolnick J. (2011) FINDSITE-metal: integrating evolutionary information and machine learning for structure-based metal-binding site prediction at the proteome level. Proteins 79, 735–751 21287609 10.1002/prot.22913 PMC3060289

[41] He W., Liang Z., Teng M. and Niu L. (2015) mFASD: a structure-based algorithm for discriminating different types of metal-binding sites. Bioinformatics 31, 1938–1944 25649619 10.1093/bioinformatics/btv044

[42] Zhao W., Xu M., Liang Z., Ding B., Niu L., Liu H. (2011) Structure-based* de novo* prediction of zinc-binding sites in proteins of unknown function. Bioinformatics 27, 1262–1268 21414989 10.1093/bioinformatics/btr133

[43] Liang M.P., Banatao D.R., Klein T.E., Brutlag D.L. and Altman R.B. (2003) WebFEATURE: an interactive web tool for identifying and visualizing functional sites on macromolecular structures. Nucleic Acids Res. 31 3324–3327 12824318 10.1093/nar/gkg553 PMC168960

[44] Ebert J.C. and Altman R.B. (2008) Robust recognition of zinc binding sites in proteins. Protein Sci. 17, 54–65 18042678 10.1110/ps.073138508 PMC2144590

[45] Wu S., Liang M.P. and Altman R.B. (2008) The SeqFEATURE library of 3D functional site models: comparison to existing methods and applications to protein function annotation. Genome Biol. 9, R8 18197987 10.1186/gb-2008-9-1-r8 PMC2395245

[46] Sodhi J.S., Bryson K., McGuffin L.J., Ward J.J., Wernisch L. and Jones D.T. (2004) Predicting metal-binding site residues in low-resolution structural models. J. Mol. Biol. 342, 307–320 15313626 10.1016/j.jmb.2004.07.019

[47] Wass M.N., Kelley L.A. and Sternberg M.J.E. (2010) 3DLigandSite: predicting ligand-binding sites using similar structures. Nucleic Acids Res. 38, W469–W473 20513649 10.1093/nar/gkq406 PMC2896164

[48] Hu X., Dong Q., Yang J. and Zhang Y. (2016) Recognizing metal and acid radical ion-binding sites by integrating *ab initio* modeling with template-based transferals. Bioinformatics 32, 3694 27899383 10.1093/bioinformatics/btw637 PMC6276887

[49] Andreini C., Cavallaro G., Rosato A. and Valasatava Y. (2013) MetalS2: a tool for the structural alignment of minimal functional sites in metal-binding proteins and nucleic acids. J. Chem. Inf. Model 53, 3064–3075 24117467 10.1021/ci400459w

[50] Valasatava Y., Rosato A., Cavallaro G. and Andreini C. (2014) MetalS(3), a database-mining tool for the identification of structurally similar metal sites. J. Biol. Inorg. Chem. 19, 937–945 24699831 10.1007/s00775-014-1128-3

[51] Andreini C., Cavallaro G. and Lorenzini S. (2012) FindGeo: a tool for determining metal coordination geometry. Bioinformatics 28, 1658–1660 22556364 10.1093/bioinformatics/bts246

[52] Zheng H., Chordia M.D., Cooper D.R., Chruszcz M., Müller P., Sheldrick G.M. (2013) Validation of metal-binding sites in macromolecular structures with the CheckMyMetal web server. Nat. Protoc. 9, 156–170 24356774 10.1038/nprot.2013.172 PMC4410975

[53] Takahashi H Metalmine [Internet] (2009) MetalMine: a database of functional metal-binding sites in proteins. [cited 2016 Aug 2]. Available from: http://metalmine.naist.jp/metalmine009/about.html

[54] Hemavathi K., Kalaivani Kaani M., Udayakumar A., Sowmiya G., Jeyakanthan J. and Sekar K. (2010) MIPS: metal interactions in protein structures. J. Appl. Cryst. 43, 196–199

[55] Hsin K.-Y., Sheng Y., Harding M.M., Taylor P. and Walkinshaw M.D. (2008) MESPEUS: a database of the geometry of metal sites in proteins. J. Appl. Cryst. 41, 963–968

[56] Degtyarenko K. and Contrino S. (2004) COM e: the ontology of bioinorganic proteins. BMC Struct. Biol. 4, 3 15113423 10.1186/1472-6807-4-3 PMC395836

[57] Choi H., Kang H. and Park H. (2011) MetLigDB: a web-based database for the identification of chemical groups to design metalloprotein inhibitors. J. Appl. Cryst. 44, 878–881

[58] Fehl C. and Davis B.G. (2016) Proteins as templates for complex synthetic metalloclusters: towards biologically programmed heterogeneous catalysis. Proc. Math. Phys. Eng. Sci. 472, 20160078 27279776 10.1098/rspa.2016.0078 PMC4893187

[59] Mann S.I., Heinisch T., Weitz A.C., Hendrich M.P., Ward T.R. and Borovik A.S. (2016) Modular artificial cupredoxins. J. Am. Chem. Soc. 138, 9073–9076 27385206 10.1021/jacs.6b05428 PMC5110120

[60] Yu F., Cangelosi V.M., Zastrow M.L., Tegoni M., Plegaria J.S., Tebo A.G. (2014) Protein design: toward functional metalloenzymes. Chem. Rev. 114, 3495–3578 24661096 10.1021/cr400458x PMC4300145

[61] Nastri F., Chino M., Maglio O., Bhagi-Damodaran A., Lu Y. and Lombardi A. (2016) Design and engineering of artificial oxygen-activating metalloenzymes. Chem. Soc. Rev. 45, 5020–5054 27341693 10.1039/c5cs00923e PMC5021598

[62] Pordea A. (2015) Metal-binding promiscuity in artificial metalloenzyme design. Curr. Opin. Chem. Biol. 25, 124–132 25603469 10.1016/j.cbpa.2014.12.035

[63] Cangelosi V.M., Deb A., Penner-Hahn J.E. and Pecoraro V.L. (2014) A *de novo* designed metalloenzyme for the hydration of CO2. Angew. Chem. Int. Ed. Engl. 53, 7900–7903 24943466 10.1002/anie.201404925 PMC4107010

[64] Dill K.A. and MacCallum J.L. (2012) The protein-folding problem, 50 years on. Science 338, 1042–1046 23180855 10.1126/science.1219021

[65] Drexler K.E. (1981) Molecular engineering: an approach to the development of general capabilities for molecular manipulation. Proc. Natl. Acad. Sci. U.S.A. 78, 5275–5278 16593078 10.1073/pnas.78.9.5275 PMC348724

[66] Pabo C. (1983) Molecular technology: designing proteins and peptides. Nature 301, 200 6823300 10.1038/301200a0

[67] Fung H.K., Welsh W.J. and Floudas C.A. (2008) Computational *de novo* peptide and protein design: rigid templates versus flexible templates. Ind. Eng. Chem. Res. 47, 993–1001

[68] Smadbeck J., Peterson M.B., Khoury G.A., Taylor M.S. and Floudas C.A. (2013) Protein WISDOM: a workbench for *in silico de* *novo* design of biomolecules. J. Vis. Exp. 25, 10.3791/50476 PMC3846368 23912941

[69] Hay B.P. and Firman T.K. (2002) HostDesigner: a program for the *de novo* structure-based design of molecular receptors with binding sites that complement metal ion guests. Inorg. Chem. 41, 5502–5512 12377046 10.1021/ic0202920

[70] Clarke N.D. and Yuan S.-M. (1995) Metal search: a computer program that helps design tetrahedral metal-binding sites. Proteins 23, 256–263 8592706 10.1002/prot.340230214

[71] Jiang X., Farid H., Pistor E. and Farid R.S. (2000) A new approach to the design of uniquely folded thermally stable proteins. Protein Sci. 9, 403–416 10716193 10.1110/ps.9.2.403 PMC2144549

[72] Liu Y. and Kuhlman B. (2006) RosettaDesign server for protein design. Nucleic Acids Res. 34, W235–W238 16845000 10.1093/nar/gkl163 PMC1538902

[73] Mitra P., Shultis D. and Zhang Y. (2013) EvoDesign: *de novo* protein design based on structural and evolutionary profiles. Nucleic Acids Res. 41, W273–W280 23671331 10.1093/nar/gkt384 PMC3692067

[74] Lauck F., Smith C.A., Friedland G.F., Humphris E.L. and Kortemme T. (2010) RosettaBackrub–a web server for flexible backbone protein structure modeling and design. Nucleic Acids Res. 38, W569–W575 20462859 10.1093/nar/gkq369 PMC2896185

[75] Fazelinia H., Cirino P.C. and Maranas C.D. (2009) OptGraft: A computational procedure for transferring a binding site onto an existing protein scaffold. Protein Sci. 18, 180–195 19177362 10.1002/pro.2 PMC2708043

[76] Chaudhury S., Lyskov S. and Gray J.J. (2010) PyRosetta: a script-based interface for implementing molecular modeling algorithms using Rosetta. Bioinformatics 26, 689–691 20061306 10.1093/bioinformatics/btq007 PMC2828115

[77] Farinas E. and Regan L. (1998) The *de novo* design of a rubredoxin-like Fe site. Protein Sci. 7, 1939–1946 9761474 10.1002/pro.5560070909 PMC2144162

[78] Klemba M., Gardner K.H., Marino S., Clarke N.D. and Regan L. (1995) Novel metal-binding proteins by design. Nat. Struct. Mol. Biol. 2, 368–373 10.1038/nsb0595-368 7664093

[79] Regan L. and Clarke N.D. (1990) A tetrahedral zinc(II)-binding site introduced into a designed protein. Biochemistry 29, 10878–10883 2271687 10.1021/bi00501a003

[80] Nanda V., Rosenblatt M.M., Osyczka A., Kono H., Getahun Z., Dutton P.L. (2005) *De novo* design of a redox-active minimal rubredoxin mimic. J. Am. Chem. Soc. 127, 5804–5805 15839675 10.1021/ja050553f

[81] Cristian L., Piotrowiak P. and Farid R.S. (2003) Mimicking photosynthesis in a computationally designed synthetic metalloprotein. J. Am. Chem. Soc. 125, 11814–11815 14505392 10.1021/ja0292142

[82] Heinisch T., Pellizzoni M., Dürrenberger M., Tinberg C.E., Köhler V., Klehr J. (2015) Improving the catalytic performance of an artificial metalloenzyme by computational design. J. Am. Chem. Soc. 137, 10414–10419 26226626 10.1021/jacs.5b06622

[83] Monnard F.W., Heinisch T., Nogueira E.S., Schirmer T. and Ward T.R. (2011) Human carbonic anhydrase II as a host for piano-stool complexes bearing a sulfonamide anchor. Chem. Commun. (Camb.) 47, 8238–8240 21706094 10.1039/c1cc10345h

[84] Gamenara D. and Dominguez de Maria P. (2014) Enantioselective imine reduction catalyzed by imine reductases and artificial metalloenzymes. Org. Biomol. Chem. 12, 2989–2992 24695640 10.1039/c3ob42205d

[85] Mills J.H., Khare S.D., Bolduc J.M., Forouhar F., Mulligan V.K., Lew S. (2013) Computational design of an unnatural amino acid dependent metalloprotein with atomic level accuracy. J. Am. Chem. Soc. 135, 13393–13399 23924187 10.1021/ja403503m PMC3863684

[86] Zanghellini A., Jiang L., Wollacott A.M., Cheng G., Meiler J., Althoff E.A. (2006) New algorithms and an *in silico* benchmark for computational enzyme design. Protein Sci. 15, 2785–2794 17132862 10.1110/ps.062353106 PMC2242439

[87] Zhu C., Zhang C., Liang H. and Lai L. (2011) Engineering a zinc binding site into the *de novo* designed protein DS119 with a βαβ structure. Protein Cell 2, 1006–1013 22231358 10.1007/s13238-011-1121-3 PMC4875247

[88] Yeung N., Lin Y.-W., Gao Y.-G., Zhao X., Russell B.S., Lei L. (2009) Rational design of a structural and functional nitric oxide reductase. Nature 462, 1079–1082 19940850 10.1038/nature08620 PMC4297211

[89] Salgado E.N., Ambroggio X.I., Brodin J.D., Lewis R.A., Kuhlman B. and Tezcan F.A. (2010) Metal templated design of protein interfaces. Proc. Natl. Acad. Sci. U.S.A. 107, 1827–1832 20080561 10.1073/pnas.0906852107 PMC2836610

[90] Rufo C.M., Moroz Y.S., Moroz O.V., Stöhr J., Smith T.A., Hu X. (2014) Short peptides self-assemble to produce catalytic amyloids. Nat. Chem. 6, 303–309 24651196 10.1038/nchem.1894 PMC3996680

[91] Tanaka T., Mizuno T., Fukui S., Hiroaki H., Oku J., Kanaori K. (2004) Two-metal ion, Ni(II) and Cu(II), binding alpha-helical coiled coil peptide. J. Am. Chem. Soc. 126, 14023–14028 15506765 10.1021/ja047945r

[92] Nanda V. and Koder R.L. (2010) Designing artificial enzymes by intuition and computation. Nat. Chem. 2, 15–24 21124375 10.1038/nchem.473 PMC3443871

[93] Zastrow M.L., Peacock A.F., Stuckey J.A. and Pecoraro V.L. (2012) Hydrolytic catalysis and structural stabilization in a designed metalloprotein. Nat. Chem. 4, 118–123 10.1038/nchem.1201 PMC3270697 22270627

[94] Zastrow M.L. and Pecoraro V.L. (2013) The influence of active site location on catalytic activity in *de novo* designed zinc metalloenzymes. J. Am. Chem. Soc. 135, 5895–5903 23516959 10.1021/ja401537t PMC3667658

[95] Tebo A.G. and Pecoraro V.L. (2015) Artificial metalloenzymes derived from three-helix bundles. Curr. Opin. Chem. Biol. 25, 65–70 25579452 10.1016/j.cbpa.2014.12.034 PMC4380837

[96] Plegaria J.S., Herrero C., Quaranta A. and Pecoraro V.L. (2016) Electron transfer activity of a *de* *novo* designed copper center in a three-helix bundle fold. Biochim. Biophys. Acta 1857, 522–530 26427552 10.1016/j.bbabio.2015.09.007 PMC5233711

[97] Key H.M., Dydio P., Clark D.S. and Hartwig J.F. (2016) Abiological catalysis by artificial haem proteins containing noble metals in place of iron. Nature 534, 534–537 27296224 10.1038/nature17968 PMC11723505

[98] Quinto T., Haussinger D., Kohler V. and Ward T.R. (2015) Artificial metalloenzymes for the diastereoselective reduction of NAD(+) to NAD(2)H. Org. Biomol. Chem. 13, 357–360 25379837 10.1039/c4ob02071e

[99] Lo C., Ringenberg M.R., Gnandt D., Wilson Y. and Ward T.R. (2011) Artificial metalloenzymes for olefin metathesis based on the biotin-(strept)avidin technology. Chem. Commun. (Camb.) 47, 12065–12067 21959544 10.1039/c1cc15004a

[100] Okamoto Y., Köhler V. and Ward T.R. (2016) An NAD(P)H-dependent artificial transfer hydrogenase for multienzymatic cascades. J. Am. Chem. Soc. 138, 5781–5784 27100673 10.1021/jacs.6b02470

[101] Shiga D., Funahashi Y., Masuda H., Kikuchi A., Noda M., Uchiyama S. (2012) Creation of a binuclear purple copper site within a *de novo* coiled-coil protein. Biochemistry 51, 7901–7907 22989113 10.1021/bi3007884

